# Supplementary material for: Implication of Opioid Receptors in the Antihypertensive Effect of a Novel Chicken Foot-Derived Peptide
Source: Biomolecules. 2020 Jul 2;10(7):992. doi: 10.3390/biom10070992 (PMC7408493; doi:10.3390/biom10070992)
Supplement: Supplementary file 1 [file biomolecules-10-00992-s001.pdf]

SUPPLEMENTARY MATERIAL

Table S1. Recovery and reproducibility of the evaluated peptide extraction methods from plasma

Methods	Recovery (%)[a]	RSD (%)[b]
SPE	92.4	2.5
TFA	48.2	28.3
TFA + SPE	37.8	26.5

SPE, plasma + solid phase extraction; TFA, plasma + trifluoroacetic acid; TFA + SPE, trifluoroacetic acid + solid phase extraction. [a] Average percent recovery of the peptides by the described method (n= 3). [b] Repeatability (RSD—relative standard deviation of determined concentration).
